# Diaqua­bis­(nitrato-κ^2^ *O*,*O*′)bis­(pyrazine-2-carboxamide-κ*N* ^4^)cadmium–pyrazine-2-carboxamide (1/2)

**DOI:** 10.1107/S1600536812028577

**Published:** 2012-06-30

**Authors:** Sadif A. Shirvan, Sara Haydari Dezfuli

**Affiliations:** a Department of Chemistry, Omidieh Branch, Islamic Azad University, Omidieh, Iran

## Abstract

In the title compound, [Cd(NO_3_)_2_(C_5_H_5_N_3_O)_2_(H_2_O)_2_]·2C_5_H_5_N_3_O, the Cd^II^ cation is located on a twofold rotation axis and is coordinated by two pyrazine-2-carboxamide ligands and two water mol­ecules and chelated by two nitrate anions in a distorted square-anti­prismatic geometry. Extensive inter­molecular N—H⋯O, N—H⋯N, O—H⋯O and O—H⋯N hydrogen bonds, as well as weak inter­molecular C—H⋯N and C—H⋯O inter­actions occur in the crystal. π–π stacking between between pyrazine rings of coordinating ligands and lattice molecules [centroid–centroid distance = 3.5669 (14) Å] may further stabilize the structure.

## Related literature

For related structures, see: Abu-Youssef *et al.* (2006 ▶); Azhdari Tehrani *et al.* (2010 ▶); Goher & Mautner (2000 ▶); Kristiansson (2002 ▶); Mir Mohammad Sadegh *et al.* (2010 ▶); Munakata *et al.* (1997 ▶); Pacigova *et al.* (2008 ▶); Shirvan & Haydari Dezfuli (2012*a* ▶,*b* ▶,*c* ▶).
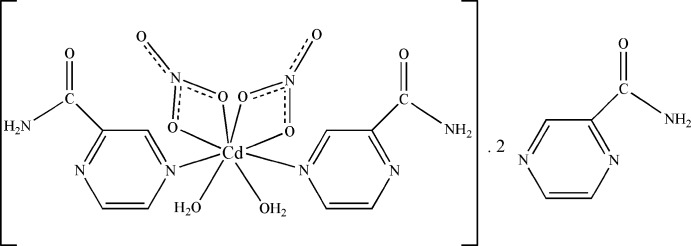


## Experimental

### 

#### Crystal data

[Cd(NO_3_)_2_(C_5_H_5_N_3_O)_2_(H_2_O)_2_]·2C_5_H_5_N_3_O
*M* *_r_* = 764.94
Monoclinic, 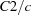

*a* = 13.5650 (5) Å
*b* = 6.7845 (3) Å
*c* = 31.2031 (11) Å
β = 95.665 (3)°
*V* = 2857.65 (19) Å^3^
*Z* = 4
Mo *K*α radiation
μ = 0.85 mm^−1^
*T* = 298 K
0.22 × 0.21 × 0.20 mm

#### Data collection

Bruker APEXII CCD area-detector diffractometer
Absorption correction: multi-scan (*SADABS*; Bruker, 2001 ▶) *T*_min_ = 0.835, *T*_max_ = 0.865
15388 measured reflections
3850 independent reflections
3099 reflections with *I* > 2σ(*I*)
*R*_int_ = 0.067

#### Refinement

*R*[*F* ^2^ > 2σ(*F* ^2^)] = 0.039
*wR*(*F* ^2^) = 0.069
*S* = 1.02
3850 reflections
221 parameters
2 restraints
H atoms treated by a mixture of independent and constrained refinement
Δρ_max_ = 0.44 e Å^−3^
Δρ_min_ = −0.51 e Å^−3^

### 

Data collection: *APEX2* (Bruker, 2007 ▶); cell refinement: *SAINT* (Bruker, 2007 ▶); data reduction: *SAINT*; program(s) used to solve structure: *SHELXTL* (Sheldrick, 2008 ▶); program(s) used to refine structure: *SHELXTL*; molecular graphics: *SHELXTL*; software used to prepare material for publication: *SHELXTL*.

## Supplementary Material

Crystal structure: contains datablock(s) I, global. DOI: 10.1107/S1600536812028577/xu5577sup1.cif

Structure factors: contains datablock(s) I. DOI: 10.1107/S1600536812028577/xu5577Isup2.hkl

Additional supplementary materials:  crystallographic information; 3D view; checkCIF report

## Figures and Tables

**Table 1:** Selected bond lengths (Å)

Cd1—O3	2.386 (2)
Cd1—O4	2.529 (2)
Cd1—O5	2.469 (2)
Cd1—N2	2.3459 (19)

**Table 2:** Hydrogen-bond geometry (Å, °)

*D*—H⋯*A*	*D*—H	H⋯*A*	*D*⋯*A*	*D*—H⋯*A*
O3—H3*D*⋯N6^i^	0.82 (1)	2.13 (1)	2.937 (3)	169 (4)
O3—H3*E*⋯O5^ii^	0.82 (3)	2.44 (3)	3.215 (4)	158 (3)
O3—H3*E*⋯O6^ii^	0.82 (3)	2.39 (3)	3.130 (3)	150 (3)
N3—H3*B*⋯O2	0.86	2.22	3.072 (3)	170
N3—H3*C*⋯O2^iii^	0.86	2.30	2.987 (3)	137
N4—H4*A*⋯N5^iv^	0.86	2.50	3.180 (3)	137
N4—H4*B*⋯O1	0.86	1.97	2.832 (3)	179
C6—H6⋯N1^iii^	0.93	2.57	3.364 (4)	143
C8—H8⋯O1^iv^	0.93	2.57	3.429 (3)	155

Symmetry codes: (i) 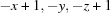; (ii) 
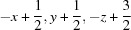
; (iii) 
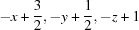
; (iv) 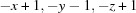.

**Fig. 1.**
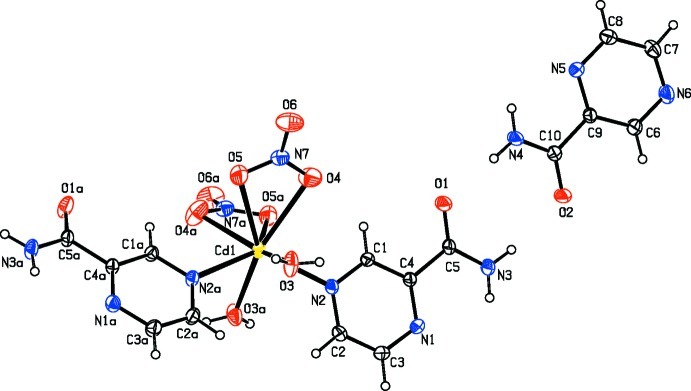
The molecular structure of the title molecule, with the atom-numbering scheme. Displacement ellipsoids are drawn at the 30% probability level. [Symmetry codes: (a) 1 - x,y,3/2 - z].

**Fig. 2.**
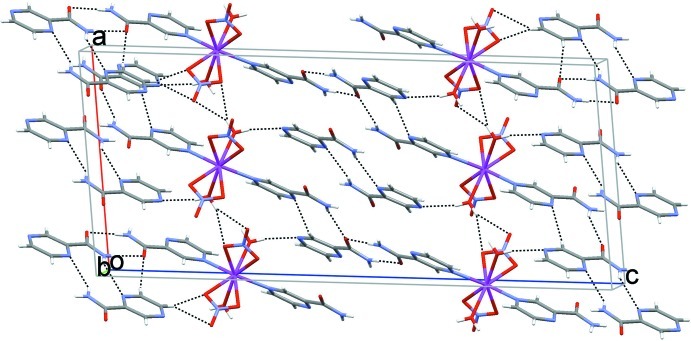
Unit-cell packing diagram for title molecule. Hydrogen bonds are shown as dashed lines.

## supplementary crystallographic information

### Comment

Pyrazine-2-carboxamide (pzc), is a good ligand, and a few complexes with pzc have been prepared, such as that of mercury (Azhdari Tehrani *et al.*, 2010; Mir Mohammad Sadegh *et al.*, 2010), vanadium (Pacigova *et al.*, 2008), manganese (Abu-Youssef *et al.*, 2006), copper (Kristiansson, 2002; Munakata *et al.*, 1997; Goher & Mautner, 2000) and zinc (Shirvan & Haydari Dezfuli, 2012*a*,*b*,*c*). Here, we report the synthesis and structure of the title compound.

The asymmetric unit of the title compound, (Fig. 1), contains one half of Cd^II^ atom, one coordinated pyrazine-2-carboxamide ligand, one water molecule, one nitrate anion and one none coordinated pyrazine-2-carboxamide. The Cd^II^ atom is eight-coordinated by two N atoms from two pyrazine-2-carboxamide ligands, two O atoms from two water molecules and four O atoms from two nitrate anions. The Cd—O and Cd—N bond lengths and angles are collected in Table 1.

In the crystal structure, intra and intermolecular N—H···O, N—H···N, O—H···O, C—H···N and C—H···O hydrogen bonds (Table 2) and π-π contacts (Fig. 2) between the pyrazine rings, *Cg*1—*Cg*2^i^ [symmetry cods: (i) 1-*X*,-Y,1-*Z*, where *Cg*1 and *Cg*2 are centroids of the rings (N1/C2—C3/N2/C1/C4) and (N5/C7—C8/N6/C6/C9), respectively] may stabilize the structure, with centroid-centroid distance of 3.5669 (14) Å.

### Experimental

A solution of pyrazine-2-carboxamide (0.50 g, 4.0 mmol) in methanol (10 ml) was added to a solution of Cd(NO_3_)_2_.4H_2_O (0.31 g, 1.0 mmol) in methanol (10 ml) and the resulting colorless solution was stirred for 15 min at room temperature. This solution was left to evaporate slowly at room temperature. After one week, colorless block crystals of the title compound were isolated (yield 0.56 g, 73.2%).

### Refinement

H atoms were positioned geometrically with C—H = 0.93 and N—H = 0.86 Å, and constrained to ride on their parent atoms, U_iso_(H) = 1.2U_eq_(N,C).

### Crystal data

**Table:**

[Cd(NO_3_)_2_(C_5_H_5_N_3_O)_2_(H_2_O)_2_]·2C_5_H_5_N_3_O	*F*(000) = 1544
*M**_r_* = 764.94	*D*_x_ = 1.778 Mg m^−^^3^
Monoclinic, *C*2/*c*	Mo *K*α radiation, λ = 0.71073 Å
Hall symbol: -C 2yc	Cell parameters from 15388 reflections
*a* = 13.5650 (5) Å	θ = 2.6–29.2°
*b* = 6.7845 (3) Å	µ = 0.85 mm^−^^1^
*c* = 31.2031 (11) Å	*T* = 298 K
β = 95.665 (3)°	Block, colorless
*V* = 2857.65 (19) Å^3^	0.22 × 0.21 × 0.20 mm
*Z* = 4	

### Data collection

**Table:**

Bruker APEXII CCD area-detector diffractometer	3850 independent reflections
Radiation source: fine-focus sealed tube	3099 reflections with *I* > 2σ(*I*)
Graphite monochromator	*R*_int_ = 0.067
ω scans	θ_max_ = 29.2°, θ_min_ = 2.6°
Absorption correction: multi-scan (*SADABS*; Bruker, 2001)	*h* = −15→18
*T*_min_ = 0.835, *T*_max_ = 0.865	*k* = −9→9
15388 measured reflections	*l* = −42→42

### Refinement

**Table:**

Refinement on *F*^2^	Primary atom site location: structure-invariant direct methods
Least-squares matrix: full	Secondary atom site location: difference Fourier map
*R*[*F*^2^ > 2σ(*F*^2^)] = 0.039	Hydrogen site location: inferred from neighbouring sites
*wR*(*F*^2^) = 0.069	H atoms treated by a mixture of independent and constrained refinement
*S* = 1.02	*w* = 1/[σ^2^(*F*_o_^2^) + (0.0281*P*)^2^] where *P* = (*F*_o_^2^ + 2*F*_c_^2^)/3
3850 reflections	(Δ/σ)_max_ = 0.006
221 parameters	Δρ_max_ = 0.44 e Å^−^^3^
2 restraints	Δρ_min_ = −0.51 e Å^−^^3^

### Special details

**Table:**

Geometry. All e.s.d.'s (except the e.s.d. in the dihedral angle between two l.s. planes) are estimated using the full covariance matrix. The cell e.s.d.'s are taken into account individually in the estimation of e.s.d.'s in distances, angles and torsion angles; correlations between e.s.d.'s in cell parameters are only used when they are defined by crystal symmetry. An approximate (isotropic) treatment of cell e.s.d.'s is used for estimating e.s.d.'s involving l.s. planes.
Refinement. Refinement of *F*^2^ against ALL reflections. The weighted *R*-factor *wR* and goodness of fit *S* are based on *F*^2^, conventional *R*-factors *R* are based on *F*, with *F* set to zero for negative *F*^2^. The threshold expression of *F*^2^ > σ(*F*^2^) is used only for calculating *R*-factors(gt) *etc*. and is not relevant to the choice of reflections for refinement. *R*-factors based on *F*^2^ are statistically about twice as large as those based on *F*, and *R*- factors based on ALL data will be even larger.

### Fractional atomic coordinates and isotropic or equivalent isotropic displacement parameters (Å^2^)

**Table:**

	*x*	*y*	*z*	*U*_iso_*/*U*_eq_	
C1	0.56965 (17)	0.2539 (3)	0.65471 (7)	0.0297 (5)	
H1	0.5493	0.1230	0.6550	0.036*	
C2	0.5882 (2)	0.5543 (3)	0.68646 (8)	0.0360 (6)	
H2	0.5799	0.6396	0.7092	0.043*	
C3	0.6311 (2)	0.6240 (4)	0.65109 (8)	0.0388 (6)	
H3	0.6524	0.7543	0.6510	0.047*	
C4	0.61084 (17)	0.3260 (3)	0.61922 (7)	0.0271 (5)	
C5	0.61752 (18)	0.1922 (3)	0.58134 (7)	0.0309 (5)	
C6	0.6697 (2)	−0.3057 (4)	0.40570 (8)	0.0409 (6)	
H6	0.6978	−0.1827	0.4018	0.049*	
C7	0.6301 (2)	−0.6120 (4)	0.38209 (9)	0.0407 (6)	
H7	0.6307	−0.7109	0.3615	0.049*	
C8	0.5894 (2)	−0.6511 (4)	0.41992 (9)	0.0401 (6)	
H8	0.5617	−0.7745	0.4237	0.048*	
C9	0.63094 (18)	−0.3443 (3)	0.44398 (7)	0.0294 (5)	
C10	0.63336 (19)	−0.1919 (3)	0.47903 (8)	0.0323 (5)	
N1	0.64298 (17)	0.5111 (3)	0.61710 (7)	0.0349 (5)	
N2	0.55839 (15)	0.3686 (3)	0.68886 (6)	0.0288 (4)	
N3	0.66404 (16)	0.2610 (3)	0.54907 (6)	0.0400 (5)	
H3B	0.6688	0.1894	0.5266	0.048*	
H3C	0.6895	0.3772	0.5506	0.048*	
N4	0.58381 (19)	−0.2357 (3)	0.51183 (7)	0.0473 (6)	
H4B	0.5827	−0.1543	0.5329	0.057*	
H4A	0.5525	−0.3458	0.5122	0.057*	
N5	0.58844 (17)	−0.5180 (3)	0.45120 (7)	0.0357 (5)	
N6	0.66844 (19)	−0.4378 (4)	0.37415 (7)	0.0457 (6)	
N7	0.35495 (17)	−0.0688 (3)	0.72058 (7)	0.0381 (5)	
O1	0.57917 (17)	0.0284 (3)	0.58187 (6)	0.0502 (5)	
O2	0.68015 (15)	−0.0373 (3)	0.47604 (6)	0.0452 (5)	
O3	0.35625 (17)	0.3987 (4)	0.72005 (7)	0.0526 (5)	
H3D	0.341 (3)	0.410 (5)	0.6940 (4)	0.068 (11)*	
H3E	0.3053 (18)	0.407 (6)	0.7322 (12)	0.085 (14)*	
O4	0.41705 (19)	−0.0161 (3)	0.69628 (8)	0.0698 (7)	
O5	0.3670 (2)	−0.0147 (3)	0.75901 (7)	0.0651 (7)	
O6	0.28421 (17)	−0.1698 (3)	0.70757 (9)	0.0702 (7)	
Cd1	0.5000	0.22584 (4)	0.7500	0.03053 (8)	

### Atomic displacement parameters (Å^2^)

**Table:**

	*U*^11^	*U*^22^	*U*^33^	*U*^12^	*U*^13^	*U*^23^
C1	0.0371 (12)	0.0264 (11)	0.0269 (10)	−0.0051 (9)	0.0093 (9)	−0.0015 (9)
C2	0.0477 (16)	0.0323 (12)	0.0292 (13)	−0.0037 (11)	0.0087 (11)	−0.0067 (9)
C3	0.0531 (17)	0.0288 (12)	0.0356 (14)	−0.0102 (11)	0.0106 (12)	−0.0035 (10)
C4	0.0290 (12)	0.0297 (11)	0.0230 (11)	−0.0047 (9)	0.0042 (9)	−0.0015 (8)
C5	0.0366 (13)	0.0343 (12)	0.0228 (11)	−0.0046 (10)	0.0078 (10)	−0.0024 (9)
C6	0.0480 (16)	0.0442 (14)	0.0325 (13)	−0.0153 (12)	0.0143 (12)	−0.0046 (11)
C7	0.0437 (16)	0.0435 (14)	0.0354 (14)	0.0006 (12)	0.0059 (12)	−0.0118 (11)
C8	0.0485 (16)	0.0320 (12)	0.0404 (15)	−0.0047 (11)	0.0071 (13)	−0.0063 (10)
C9	0.0299 (12)	0.0347 (11)	0.0238 (11)	−0.0058 (9)	0.0042 (10)	−0.0018 (9)
C10	0.0366 (13)	0.0333 (12)	0.0275 (12)	−0.0060 (10)	0.0047 (10)	−0.0025 (9)
N1	0.0441 (13)	0.0321 (10)	0.0298 (11)	−0.0089 (9)	0.0102 (10)	0.0020 (8)
N2	0.0314 (11)	0.0322 (10)	0.0237 (10)	−0.0026 (8)	0.0072 (8)	−0.0028 (7)
N3	0.0525 (13)	0.0427 (12)	0.0274 (9)	−0.0133 (10)	0.0171 (9)	−0.0060 (9)
N4	0.0688 (15)	0.0409 (12)	0.0364 (11)	−0.0256 (11)	0.0254 (11)	−0.0140 (10)
N5	0.0453 (13)	0.0313 (10)	0.0318 (11)	−0.0063 (9)	0.0096 (10)	−0.0002 (8)
N6	0.0517 (15)	0.0558 (14)	0.0316 (12)	−0.0092 (11)	0.0136 (11)	−0.0071 (10)
N7	0.0377 (13)	0.0308 (11)	0.0468 (14)	−0.0010 (9)	0.0088 (11)	−0.0010 (9)
O1	0.0752 (15)	0.0408 (10)	0.0390 (11)	−0.0237 (10)	0.0281 (10)	−0.0141 (8)
O2	0.0569 (13)	0.0402 (10)	0.0411 (11)	−0.0225 (9)	0.0175 (10)	−0.0087 (8)
O3	0.0475 (14)	0.0815 (15)	0.0305 (11)	0.0188 (11)	0.0128 (10)	0.0083 (10)
O4	0.0758 (17)	0.0646 (14)	0.0773 (17)	−0.0271 (12)	0.0495 (14)	−0.0193 (12)
O5	0.107 (2)	0.0472 (12)	0.0426 (13)	−0.0008 (12)	0.0159 (13)	−0.0011 (9)
O6	0.0452 (13)	0.0619 (14)	0.103 (2)	−0.0182 (11)	0.0033 (13)	−0.0088 (13)
Cd1	0.03867 (15)	0.03185 (13)	0.02309 (12)	0.000	0.01321 (10)	0.000

### Geometric parameters (Å, º)

**Table:**

Cd1—O3	2.386 (2)	N3—H3C	0.8600
Cd1—O4	2.529 (2)	N4—C10	1.313 (3)
Cd1—O5	2.469 (2)	N5—C8	1.331 (3)
Cd1—N2	2.3459 (19)	N5—C9	1.341 (3)
Cd1—O3^i^	2.386 (2)	N6—C7	1.324 (4)
Cd1—O4^i^	2.529 (2)	N6—C6	1.330 (4)
Cd1—O5^i^	2.469 (2)	N4—H4B	0.8600
Cd1—N2^i^	2.3459 (19)	N4—H4A	0.8600
O1—C5	1.228 (3)	C1—C4	1.378 (3)
O4—N7	1.240 (3)	C2—C3	1.381 (4)
O5—N7	1.249 (3)	C4—C5	1.500 (3)
O6—N7	1.216 (3)	C1—H1	0.9300
O3—H3D	0.822 (14)	C2—H2	0.9300
O3—H3E	0.82 (3)	C3—H3	0.9300
O2—C10	1.234 (3)	C6—C9	1.377 (3)
N1—C3	1.331 (3)	C7—C8	1.377 (4)
N1—C4	1.333 (3)	C9—C10	1.503 (3)
N2—C2	1.328 (3)	C6—H6	0.9300
N2—C1	1.340 (3)	C7—H7	0.9300
N3—C5	1.325 (3)	C8—H8	0.9300
N3—H3B	0.8600		
			
O3—Cd1—O4	76.50 (8)	O5—N7—O6	120.7 (3)
O3—Cd1—O5	77.96 (8)	H3B—N3—H3C	120.00
O3—Cd1—N2	78.87 (7)	C5—N3—H3B	120.00
O3—Cd1—O3^i^	121.11 (9)	C5—N3—H3C	120.00
O3—Cd1—O4^i^	150.64 (8)	C8—N5—C9	116.0 (2)
O3—Cd1—O5^i^	149.14 (8)	C6—N6—C7	116.1 (2)
O3—Cd1—N2^i^	77.71 (7)	C10—N4—H4B	120.00
O4—Cd1—O5	50.53 (8)	C10—N4—H4A	120.00
O4—Cd1—N2	83.85 (7)	H4A—N4—H4B	120.00
O3^i^—Cd1—O4	150.64 (8)	N2—C1—C4	121.31 (19)
O4—Cd1—O4^i^	99.08 (7)	N2—C2—C3	121.7 (2)
O4—Cd1—O5^i^	77.17 (8)	N1—C3—C2	122.2 (2)
O4—Cd1—N2^i^	130.02 (8)	C1—C4—C5	118.62 (19)
O5—Cd1—N2	132.42 (7)	N1—C4—C5	119.0 (2)
O3^i^—Cd1—O5	149.14 (8)	N1—C4—C1	122.3 (2)
O4^i^—Cd1—O5	77.17 (8)	N3—C5—C4	117.13 (19)
O5—Cd1—O5^i^	97.26 (8)	O1—C5—C4	118.6 (2)
O5—Cd1—N2^i^	82.60 (7)	O1—C5—N3	124.3 (2)
O3^i^—Cd1—N2	77.71 (7)	C4—C1—H1	119.00
O4^i^—Cd1—N2	130.02 (8)	N2—C1—H1	119.00
O5^i^—Cd1—N2	82.60 (7)	C3—C2—H2	119.00
N2—Cd1—N2^i^	131.23 (7)	N2—C2—H2	119.00
O3^i^—Cd1—O4^i^	76.50 (8)	C2—C3—H3	119.00
O3^i^—Cd1—O5^i^	77.96 (8)	N1—C3—H3	119.00
O3^i^—Cd1—N2^i^	78.87 (7)	N6—C6—C9	122.4 (2)
O4^i^—Cd1—O5^i^	50.53 (8)	N6—C7—C8	122.0 (3)
O4^i^—Cd1—N2^i^	83.85 (7)	N5—C8—C7	122.1 (2)
O5^i^—Cd1—N2^i^	132.42 (7)	C6—C9—C10	121.2 (2)
Cd1—O4—N7	93.81 (16)	N5—C9—C6	121.4 (2)
Cd1—O5—N7	96.47 (16)	N5—C9—C10	117.5 (2)
H3D—O3—H3E	108 (4)	O2—C10—N4	123.9 (2)
Cd1—O3—H3D	123 (3)	O2—C10—C9	120.3 (2)
Cd1—O3—H3E	123 (3)	N4—C10—C9	115.8 (2)
C3—N1—C4	115.9 (2)	N6—C6—H6	119.00
C1—N2—C2	116.5 (2)	C9—C6—H6	119.00
Cd1—N2—C1	118.75 (15)	N6—C7—H7	119.00
Cd1—N2—C2	124.53 (16)	C8—C7—H7	119.00
O4—N7—O6	121.3 (2)	N5—C8—H8	119.00
O4—N7—O5	118.0 (2)	C7—C8—H8	119.00
			
O3—Cd1—O4—N7	−79.22 (16)	Cd1—O4—N7—O6	169.2 (2)
O5—Cd1—O4—N7	6.18 (14)	Cd1—O5—N7—O4	11.0 (2)
N2—Cd1—O4—N7	−159.21 (16)	Cd1—O5—N7—O6	−168.8 (2)
O3^i^—Cd1—O4—N7	149.72 (17)	C4—N1—C3—C2	0.0 (4)
O4^i^—Cd1—O4—N7	71.16 (16)	C3—N1—C4—C1	−1.3 (4)
O5^i^—Cd1—O4—N7	117.01 (16)	C3—N1—C4—C5	177.3 (2)
N2^i^—Cd1—O4—N7	−18.40 (19)	Cd1—N2—C2—C3	173.36 (19)
O3—Cd1—O5—N7	76.16 (16)	C1—N2—C2—C3	−1.7 (4)
O4—Cd1—O5—N7	−6.16 (14)	Cd1—N2—C1—C4	−174.91 (17)
N2—Cd1—O5—N7	13.7 (2)	C2—N2—C1—C4	0.5 (3)
O3^i^—Cd1—O5—N7	−151.55 (16)	C8—N5—C9—C10	−178.6 (2)
O4^i^—Cd1—O5—N7	−119.56 (17)	C9—N5—C8—C7	−1.0 (4)
O5^i^—Cd1—O5—N7	−72.90 (16)	C8—N5—C9—C6	2.3 (4)
N2^i^—Cd1—O5—N7	155.10 (17)	C6—N6—C7—C8	3.1 (4)
O3—Cd1—N2—C1	−104.80 (18)	C7—N6—C6—C9	−1.9 (4)
O4—Cd1—N2—C1	−27.40 (17)	N2—C1—C4—C5	−177.5 (2)
O5—Cd1—N2—C1	−42.7 (2)	N2—C1—C4—N1	1.1 (4)
O3^i^—Cd1—N2—C1	129.63 (18)	N2—C2—C3—N1	1.6 (4)
O4^i^—Cd1—N2—C1	69.30 (19)	N1—C4—C5—N3	6.2 (3)
O5^i^—Cd1—N2—C1	50.41 (17)	C1—C4—C5—O1	5.6 (3)
N2^i^—Cd1—N2—C1	−167.35 (15)	N1—C4—C5—O1	−173.1 (2)
O3—Cd1—N2—C2	80.2 (2)	C1—C4—C5—N3	−175.1 (2)
O4—Cd1—N2—C2	157.6 (2)	N6—C6—C9—N5	−0.9 (4)
O5—Cd1—N2—C2	142.32 (19)	N6—C6—C9—C10	−179.9 (2)
O3^i^—Cd1—N2—C2	−45.4 (2)	N6—C7—C8—N5	−1.8 (4)
O4^i^—Cd1—N2—C2	−105.7 (2)	N5—C9—C10—O2	172.7 (2)
O5^i^—Cd1—N2—C2	−124.6 (2)	N5—C9—C10—N4	−7.1 (3)
N2^i^—Cd1—N2—C2	17.7 (2)	C6—C9—C10—O2	−8.2 (4)
Cd1—O4—N7—O5	−10.7 (2)	C6—C9—C10—N4	172.0 (2)

Symmetry code: (i) −*x*+1, *y*, −*z*+3/2.

### Hydrogen-bond geometry (Å, º)

**Table:**

*D*—H···*A*	*D*—H	H···*A*	*D*···*A*	*D*—H···*A*
O3—H3*D*···N6^ii^	0.82 (1)	2.13 (1)	2.937 (3)	169 (4)
O3—H3*E*···O5^iii^	0.82 (3)	2.44 (3)	3.215 (4)	158 (3)
O3—H3*E*···O6^iii^	0.82 (3)	2.39 (3)	3.130 (3)	150 (3)
N3—H3*B*···O2	0.86	2.22	3.072 (3)	170
N3—H3*C*···O2^iv^	0.86	2.30	2.987 (3)	137
N4—H4*A*···N5^v^	0.86	2.50	3.180 (3)	137
N4—H4*B*···O1	0.86	1.97	2.832 (3)	179
C6—H6···N1^iv^	0.93	2.57	3.364 (4)	143
C8—H8···O1^v^	0.93	2.57	3.429 (3)	155

Symmetry codes: (ii) −*x*+1, −*y*, −*z*+1; (iii) −*x*+1/2, *y*+1/2, −*z*+3/2; (iv) −*x*+3/2, −*y*+1/2, −*z*+1; (v) −*x*+1, −*y*−1, −*z*+1.
